# Evaluation of Olive Fruit Lipoxygenase Extraction Protocols on 9- and 13-*Z*,*E*-HPODE Formation

**DOI:** 10.3390/molecules21040506

**Published:** 2016-04-20

**Authors:** Barbara Soldo, Matilda Šprung, Gloria Mušac, Maja Pavela-Vrančić, Ivica Ljubenkov

**Affiliations:** Faculty of Science, Department of Chemistry, R. Boškovića 33, Split 21 000, Croatia; barbara@pmfst.hr (B.S.); msprung@pmfst.hr (M.Š.); Glo.bilic@gmail.com (G.M.); pavela@pmfst.hr (M.P.-V.)

**Keywords:** olive fruit, LOX, extraction protocols, enzyme activity, olive oil aroma

## Abstract

In plant tissues, enzymes implicated in the lipoxygenase (LOX) pathway are responsible for the hydroperoxydation of polyunsaturated fatty acids, ultimately leading to the production of small chemical species involved in several physiological processes. During industrial olive oil production, these enzymes are activated upon crushing and grinding of olive fruit tissue, subsequently leading to the synthesis of volatile compounds responsible for the positive aroma and flavor of the oil. An investigation of LOX activity during olive fruit ripening and malaxation could assist in the production of oils with favorable aroma and taste. Therefore, a reliable method for olive LOX purification is crucial. Here we report a critical review of six LOX extraction protocols, two of which have shown minimum enzyme activity, possibly leading to misconceptions in the interpretation of experimental data. Future research concerning olive LOX should employ extraction methods that preserve enzyme activity.

## 1. Introduction

Recent popularity of olive oil is a result of expanding interest in the Mediterranean type of diet (MedDiet) which was proved to be one of the healthiest diet types. The beneficial effect of olive oil on human health could be attributed to its chemical composition whereby monounsaturated fatty acids (MUFA) and phenolic compounds have a paramount role. Some studies have shown that MUFA may have a protective role against neurodegenerative diseases, such as age-related cognitive decline and Alzheimer’s disease. In addition, phenolic compounds have antioxidant and anti-inflammatory properties which, together with volatile compounds, affect sensory attributes of olive oil [1].

Volatile compounds contributing to positive aroma perceptions of olive oil are small molecules produced by enzymes belonging to the lipoxygenase (LOX) pathway. During olive oil production, this pathway is initiated by mechanical fragmentation of olive fruit tissue, and continues during malaxation of olive paste. Enzymes belonging to the LOX pathway perform a series of reactions, during which polyunsaturated fatty acids are oxidized and cleaved, ultimately leading to the production of esters [2]. Besides the positive effect on olive oil aroma, chemical compounds released by the action of LOX enzymes are implicated in various physiological processes important for proper plant growth and development [3]. Sensory defects in olive oil aroma occur in the case of contamination by microbial enzymes or as a consequence of fatty acid autooxidation, leading to a faulty aroma and taste [2]. Proper olive fruit processing and oil storage is thus crucial for both the flavor and the overall quality of the oil produced [4].

Lypooxigenases are non-heme iron containing enzymes that catalyze the first step in the LOX pathway, namely the hydroperoxidation of polyunsaturated fatty acids [5]. Most common substrates for LOX are linoleic (LA) and linolenic acid (LnA), whereby, depending on the enzyme’s regiospecificity, two products, (±)9-hydroperoxy-10*E*,12*Z*-octadecadienoic acid (9-*Z*,*E*-HPOD) and (±)13-hydroperoxy-9*Z*,11*E*-octadecadienoic acid (13-*Z*,*E*-HPOD), are formed. These products are afterwards cleaved by hydroperoxide lyase, the following enzyme in the LOX pathway, producing corresponding aldehydes, which are subsequently reduced to alcohols and esterified to esters [2].

Due to the key role that LOX has in the development of a desirable aroma in olive oils, various factors influencing enzyme activity such as variety, ripening stage and climate have been explored [2,6,7,8,9,10,11,12,13,14]. Results obtained in these studies have a potential application in the production of oils with favorable green and fruity sensory notes [7]. In order to characterize factors influencing LOX activity, an extraction procedure that preserves enzyme activity is of fundamental importance for experimental data collection and interpretation. Here we report an analysis of six protocols for LOX isolation and the effect that these procedures have on product formation, 9- and 13-hydroperoxy fatty acid.

## 2. Results and Discussion

### Determination of LOX Activity

Despite numerous data available for plant LOXs, information about olive LOX is scarce due to difficulties in enzyme purification [11]. These difficulties could be explained by a high concentration of phenols in the olive fruit, which can react with proteins, changing their properties and affecting their solubility. Moreover, hydrophobicity of LOX may cause protein aggregation, making the extraction process even more demanding [7]. Nonetheless, several research groups have successfully isolated olive LOX utilizing various modified procedures, independently described by Georgalaki and Donaire [15,16]. The purpose of this study was to determine the extent to which those extraction procedures affect enzyme activity. This information could be valuable to researchers interested in improving olive oil aroma by correlating different factors, such as ripening stage, variety and climate, influencing LOX enzyme activity.

The LOX extraction process was performed according to previously published procedures [6,7,8,12,13,14], and the total protein concentration was determined by the Bradford method using bovine serum albumin (BSA) as standard (Table 1).

The total amount of isolated protein ranged between 200–1600 μg·mL^−1^. The lowest protein concentration was obtained from the heavy membrane (4B) and microsomal fractions (4D) using protocol 4.

**Figure 1:**
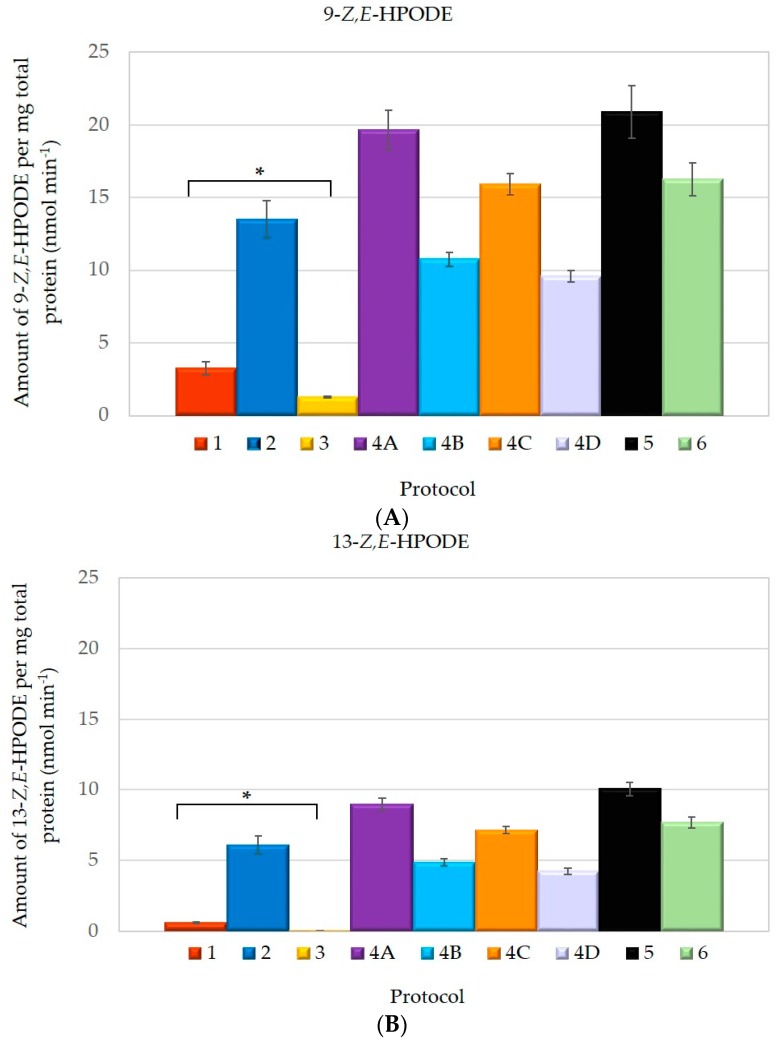
Lipoxygenase (LOX) activity calculated from the amount of: (**A**) 9-*Z*,*E*-HPODE and (**B**) 13-*Z*,*E*-HPODE, as determined by RP-HPLC with depicted bars representing * *p* << 0.05 (Anova, single factor and Scheffe test).

In order to estimate the effect of the LOX extraction procedure on enzyme activity, the concentration of 9-*Z*,*E*-HPODE and 13-*Z*,*E*-HPODE, following a 30-min incubation period, was determined by RP-HPLC. The results depicted in Figure 1A,B represent the average amount of reaction products from three independent measurements.

LOX activity, determined in our study is lower then in the original papers. This can be attributed to the variability of Croatian indigenous olive cultivar or to different olive fruit ripening stages. Additionally, our results clearly indicate that LOX predominantly produces 9-*Z*,*E*-HPODE, which is almost two times more abundant than 13-*Z*,*E*-HPODE (Table 2). This HPODE ratio was previously reported by Palmieri-Thiers *et al.* [17].

**Figure 2:**
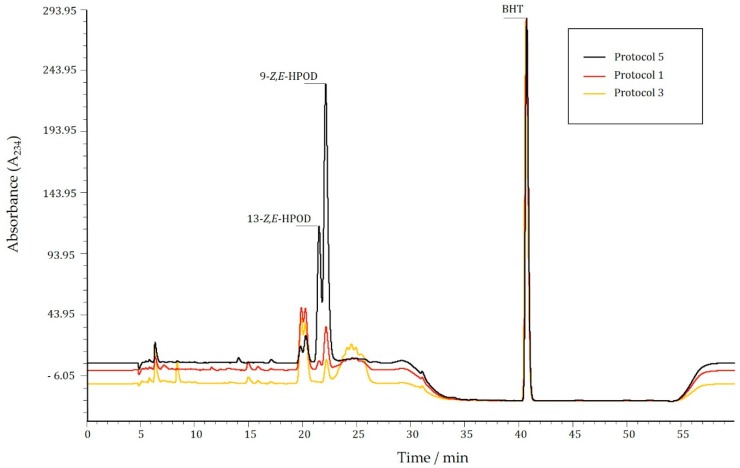
Overlaid representation of chromatograms of LOX product formation obtained by applying protocol 1, 3 and 5.

However, the most striking observation was the reduced amount of both products synthesized by LOX when using protocol 1 and 3. Although LOX extracted using protocol 1 appeared to be more active than the one obtained by protocol 3, both resulted in a significantly lower product yield compared to other tested procedures. For comparison, chromatograms of LOX product formation, following protocol 1, 3 and 5, displaying the lowest and highest enzyme activity, are represented in Figure 2.

One reason for this could be the amount of polyvinyl polypyrrolidone (PVPP), which in protocol 1 and 3, is less than 5% (*w*/*v*), compared to protocol 5 where 10% (*w*/*v*) of PVPP is used. The role of PVPP is to neutralize phenols, which otherwise inhibit LOX, consequently leading to lower enzyme activity [18], as shown with soybean lipoxygenase, proven to be inhibited by lipophylic- and hydrophylic phenolic compounds [19]. In addition, protocol 1 and 3 omitted phenylmethylsulfonyl fluoride (PMSF), an inhibitor of serine proteases. Once released, proteases could degrade LOX, contributing to lower enzyme activity encountered in LOX samples obtained applying these protocols. Protocol 6 also omits the use of PMSF but, on the contrary, has higher LOX activity most probably due to ammonium-sulphate precipitation [20].

When product formation by LOX obtained using protocol 1 and 3 was determined spectrophotometrically, it displayed, respectively, an almost 20 and 100 times higher value then the one detected by RP-HPLC (Table 3). In contrast, the level of product formation obtained using protocol 5 displayed the same value irrespective of the method. Samples extracted by protocol 1 and 3 contain impurities that could give false positive spectrophotometric measurements. Impurities can be seen in chromatograms shown in Figure 2, as a broad peak following the signal for 9-*Z*,*E*-HPODE in both sample 1 and 3. When shorter incubation period was tested (5 min), the same amount of impurities were noticed, implying that, most likely, this band is not a result of enzyme byproduct, but rather some impurity that retarded during the isolation process. Both methods, RP-HPLC and spectrophotometry, detect reaction products at a wavelength of 234 nm (A_234_), where other chemical species with conjugated double bonds could also absorb.

Taking into account our results, it is evident that two of the reported enzyme isolation procedures are not a suitable choice for LOX investigation. These protocols result in protein extracts that contain a lower amount of desirable reaction products. Therefore, if protocols 1 and 3 are to be used, spectrophotometry has to be complemented by RP-HPLC analysis in order to avoid false results regarding the level of 9-*Z*,*E*-HPODE and 13-*Z*,*E*-HPODE production. This, in turn, should help in bringing accurate conclusions about factors influencing 9-*Z*,*E*-HPODE and 13-*Z*,*E*-HPODE synthesis, with potential application in the production of oils with a favorable aroma and taste.

## 3. Materials and Methods

### 3.1. Materials

9-*Z*,*E*- and 13-*Z*,*E*-hydroperoxides were purchased from Cayman Chemical (Cayman Europe, Tallinn, Estonia). Linoleic acid (LA), butylhydroxytoluene (BHT) and all other chemicals used in this study were obtained from Sigma-Aldrich (Sigma-Aldrich, Taufkirchen, Germany)*.*

### 3.2. Plant Material

Fruits from *Olea europea L.* “Oblica” cultivar were sampled from a nearby olive-grove in Kaštela, Croatia, positioned at 43°33′40.3″N;16°22′23.3″E. The fruits were handpicked in a random manner, comprising the whole perimeter of the tree at different heights and depths of the branches. The ripeness index (RI) was determined evaluating the parameters described by Uceda *et al.* and calculated to be 2.07 [21]. Upon picking, samples were immediately stored at −80 °C.

### 3.3. Protein Extraction Procedures

LOX extraction was performed according to previously published protocols [6,7,8,12,13,14]. Olive fruits were weighted, and the olive pulp was homogenized in an extraction buffer during 4 cycles of 30 s, with a 1 min pause, on ice at 15,000 rpm’s with Polytron PT 1600 E (Kinematica, Eschbach, Germany). The homogenizate was filtered through one layer of Miracloth (Millipore, Darmstadt, Germany) and centrifuged (Table 4).

Protocols 1 and 3 complete the extraction process by the centrifugation step depicted in Table 4. Protocol 5 includes an additional centrifugation step at 10,000× *g*, for 10 min at 4 °C. In each case, the obtained pellets are discarded and supernatants, containing LOX, are further used for the enzyme activity assay. In protocol 2 the pellet is retained and resuspended in 25 mM HEPES buffer (pH 7.5) containing 10% glycerol (*v*/*v*). Protocol 6 is the only protocol where proteins from the supernatant were precipitated with 75% ammonium sulphate (*w*/*v*) and obtained by centrifugation at 20,000× *g*, for 90 min. Protein pellets were further resuspended in 50 mM sodium phosphate buffer (pH 6.8) and dialyzed overnight.

Differential centrifugation was carried out only in protocol 4. The filtered homogenizate was first centrifuged at 1000× *g* for 5 min, then at 28,000× *g* for 12 min, and finally at 100,000× *g* for one hour at 4 °C. Only the pellet from the first centrifugation step (1000× *g*) was discarded. Pellets (resuspended in 20 mM Tris buffer, pH 7.5 containing 10 mM histidine) and supernatants from other centrifugation steps (28,000 and 100,000× *g*) were further used for enzyme activity measurements. The concentration of all protein extracts was determined by the Bradford method [22].

### 3.4. Preparation of Linoleic Acid Emulsion

Linoleic acid (LA) was prepared following the method described by Axelrod *et al.* [23]. LA (25 mM) was emulgated in a water containing 1.28% Tween-20 and 30 mM NaOH. In order to eliminate dissolved oxygen, water was previously treated under nitrogen flow.

### 3.5. Determination of LOX Activity

#### 3.5.1. Synthesis and Extraction of Hydroperoxides

Synthesis and extraction of hydroperoxides was performed by modification of previously published procedure [14]. The enzyme mixture (2.5 mL) containing 0.1 M MES buffer (pH 6.0), protein extract (100 µg·mL^−1^) and linoleic acid (250 µm) was stirred for 30 min at 27 °C. The reaction was stopped by addition of HCl (1 M) to pH 2. The reaction products, 9-*Z*,*E*-HPOD and 13-*Z*,*E*-HPOD, were extracted in three successive steps, first two with 5 mL and the last one with 2.5 mL of a hexane:isopropanol solution (95:5 *v*/*v*). In addition, the extraction solution contained BHT antioxidant (0.22 mM) as an internal standard that absorbs at the detection wavelength of 234 nm, and does not interfere with LOX reaction products. Pooled extracts were further evaporated under nitrogen flow and resuspended in 200–600 µL of an acetonitrile:water mixture (67:33 *v*/*v*).

#### 3.5.2. RP-HPLC Analysis

RP-HPLC analysis was performed following slightly modified procedure described by Patui *et al.* [14]. The reaction products were separated by RP-HPLC (Perkin Elmer Series 200, Perkin Elmer, Waltham, MA, USA) on two Zorbax Eclipse XDB-C18 columns (5 µm, 4.6 mm × 250 mm and 5 µm, 4.6 mm × 150 mm, Agilent, Santa Clara, CA, USA) connected in series, using 0.25% acetic acid as solvent A and acetonitrile as solvent B, at 35 °C. After injecting 10 μL of sample, the following instrument set up parameters were applied: 0–22 min 37% solvent A, 63% solvent B; 22–47 min 20% solvent A, 80% solvent B; 47–60 min 37% solvent A and 80% solvent B at a flow rate of 0.8 mL·min^−1^. LOX reaction products were detected at 234 nm by a UV-Vis detector (Perkin Elmer Series 200) and quantified using calibration curves of pure standards. The calibration range was 0.5–64.87 μg·mL^−1^ of for (±)9-*Z*,*E*-HPOD and 0.25-35.14 μg·mL^−1^ of for (±)13-*Z*,*E*-HPOD, with *R*^2^ 0.9991 and 0.9999, respectively. Limit of detection (LOD) and limit of quantification (LOQ) for each of the reaction products is given in Table 5 below:

#### 3.5.3. Spectrophotometric Analysis

LOX activity was measured spectrophotometrically (Perkin Elmer Lambda Bio 40) at 27 °C as described in [6,7,8,12,13,14]. The increase in absorbance was recorded for 5 min and the formation of reaction products was detected at 234 nm. The reaction mixture (1 mL) contained 0.1 M MES buffer (pH 6.0), linoleic acid (250 µm) and protein extract (5 µg·mL^−1^).

### 3.6. Statistical Analysis

Microsoft Excel ANOVA single factor tool was used for the analysis of 9- and 13-*Z*,*E*-HPODE amount in all six tested protocols. In order to identify LOX extraction protocols that show the greatest difference in mean values. The Scheffe test was additionally performed.

## 4. Conclusions

The present study explores the effect of six previously reported olive LOX extraction protocols on 9- and 13-*Z*,*E*-HPODE formation. Our results point at a significantly lower amount of both HPODE products in LOX protein extracts prepared according to protocols 1 and 3. Not only does LOX prepared by these protocols have low product yield, but it also contains impurities that could interfere with spectrophotometric measurements. Therefore, if detection of olive LOX HPODE products is determined at 234 nm by spectrophotometry, confirmation by other selective methods, like RP-HPLC, is recommended. Otherwise, spectrophotometric measurements could lead to misinterpretation of LOX activity data.

## Figures and Tables

**Table 1:** Total protein concentration measured by the Bradford method using bovine serum albumin (BSA) as standard.

Reference	Protocol	Fraction	Protein Concentration/μg·mL^−1^
[8]	1		1044.40 ± 23.04
[6]	2		1026.15 ± 37.43
[7]	3		548.72 ± 20.05
[14]	4		
		A	1508.69 ± 45.01
		B	340.12 ± 25.64
		C	896.60 ± 5.96
		D	248.33 ± 15.45
[13]	5		1190.45 ± 50.66
[12]	6		970.78 ± 37.18

**Table 2:** 9- and 13-HPODE ratio in tested protocols.

Protocol	1	2	3	4A	4B	4C	4D	5	6
9-/13-*Z*,*E*-HPODE	5.47	2.22	49.85	2.19	2.21	2.23	2.26	2.08	2.12

**Table 3:** LOX specific activity determined by RP-HPLC and spectrophotometrically at 27 °C. The results represent an average from three independent measurements. The significantly lower amount of the reaction products is depicted with * *p* << 0.05 (Anova single factor and Scheffe test).

		RP-HPLC		Spectrophotometry
Reference	Protocol	Amount of Product Per mg of Total Protein (nmol·min^−1^)		Amount of Product Per mg of Total Protein (nmol·min^−1^)
		9-*Z*,*E*-HPODE	13-*Z*,*E*-HPODE	
[8]	1	3.23 ± 0.44 *	0.59 ± 0.04 *	84.08 ± 1.11
[7]	3	1.29 ± 0.06 *	0.03 ± 0.003 *	121.12 ± 3.50
[13]	5	20.89 ± 1.82	10.05 ± 0.49	37.17 ± 0.56

**Table 4:** Description of protein extraction procedures.

Protocol	1	2	3	4	5	6
Ref	[8]	[6]	[7]	[14]	[13]	[12]
Olive pulp mass (g)	10	10	10	15	10	10
Volume of Extraction buffer (mL)	40	50	50	90	40	40
Extraction buffer composition	50 mM Na_2_PO_4_ buffer, pH 6.8	50 mM HEPES	50 mM Na_2_PO_4_ buffer, pH 6.8	50 mM HEPES/KOH, pH 7.5	100 mM Na_2_PO_4_ buffer, pH 6.7	50 mM Na_2_PO_4_ buffer, pH 6.8
	0.2 mM EDTA	pH 7.5	5 mM EDTA	5 mM EDTA	1 mM EDTA	0.2 mM EDTA
	0.3 mM DTT	5 mM EDTA	3 mM DTT	3 mM DTT	/	0.3 mM DTT
	1 g PVPP	3 mM DTT	1 g PVPP	4.5 g PVPP	2 g PVPP	5 g PVPP
	(**2**% *w*/*v*)	5 g PVPP	(**2**% *w*/*v*)	(**5**% *w*/*v*)	(**5**% *w*/*v*)	(**12.5**% *w*/*v*)
	0.2% Triton X-100	(**10**% *w*/*v*)	0.12% Triton X-100	/	0.1% Triton X-100	0.1% Triton X-100
	/	/	20 mM KCl	20 mM KCl	/	/
	10 mM Na_2_S_2_O_7_	20 mM KCl	10 mM Na_2_S_2_O_7_	/	/	10 mM Na_2_S_2_O_7_
	/	/	/	2 mM MgCl_2_	/	/
	/	2 mM MgCl_2_	/	7 mM β-ME	/	/
	/	7 mM β-ME	/	0.1% ascorbate	/	/
	/	0.1% ascorbate	/	10% glycerol	/	/
	/	10% glycerol	/	/	/	/
	/	330 mM sorbitol	/	10 mM His	/	/
	/	/	/	0.25 M sucrose	/	/
	/	/	/	0.1 mM PMSF	0.1 mM PMSF	/
	/	/	/	0.1 mM benzamidine	/	/
	/	/	/	5 mM α-aminocaprioic acid	5 mM α-aminocaprioic acid	/
Homogenization	4 cycles, 30 s and 1 min of pause at 4 °C					
Centrifugation	10,000× *g*, 10 min, 4 °C	40,000× *g*, 20 min,	13,000× *g*, 20 min,	Differential	27,000× *g*, 20 min	27,000× *g*, 30 min,
		4 °C	4 °C		4 °C	4 °C

**Table 5:** Limits of detection (LOD) and quatification (LOQ) for 9-*Z*,*E*-HPODE and 13-*Z*,*E*-HPODE.

Limits of Detection and Quantifiction	9-*Z*,*E*-HPOD	13-*Z*,*E*-HPOD
LOD	0.016	0.017
LOQ	0.047	0.050

## Abbreviations

The following abbreviations are used in this manuscript:

LOX: Lipoxygenase
LA: Linoleic acid
LnA: Linolenic acid
9-*E*,*Z*-HPOD: (±)9-hydroperoxy-10*E*,12*Z*-octadecadienoic acid
13-*Z*,*E*-HPOD: (±)13-hydroperoxy-9*Z*,11*E*-octadecadienoic acid
BSA: Bovine serum albumin
RP-HPLC: reverse-phase high pressure liquid chromatography
PVPP: Polyvinyl polypyrrolidone
PMSF: Phenylmethylsulfonyl fluoride
HEPES: 4-(2-hydroxyethyl)-1-piperazineethanesulfonic acid
MES: 2-(*N*-morpholino)ethanesulfonic acid
BHT: Butylated hydroxytoluene
A_234_: Absorbance at 234 nm
